# Supplementary material for: Rapid evolution of the primate larynx?
Source: PLoS Biol. 2020 Aug 11;18(8):e3000764. doi: 10.1371/journal.pbio.3000764 (PMC7418954; doi:10.1371/journal.pbio.3000764)
Supplement: S1 Text — (DOCX) [file pbio.3000764.s001.docx]

**Body mass results**

Weights were not available for the majority of our specimens. In most cases “Weight” was given as “PM’d” in the zoo records that accompanied specimens, indicating that a post-mortem autopsy had been performed, presumably without first taking a weight. The analyses using body mass presented below are thus based on species’ averages (from references 46 and 47) rather than the specimen-specific body length measures used in the main text. This, together with the fact that body masses in the zoo often differ substantially from those in the wild, is the reason that we chose length as the main proxy of body size.

*PANCOVA analysis*. Comparison of the larynx size vs. body mass (base10 log) relationships between primates and carnivorans revealed similar slopes (*β_prim_* =1.26 vs. *β_carn_* =1.44, *F*_3,54_ =0.09, *P* =0.767), but significantly different intercepts (*α_prim_* =-0.87 vs. *α_carn_* =-1.67, *F*_3,54_ =17.655, *P* <0.001).

*Co-variate analyses*. No significant effects of the co-variates, or their interactions with body mass (BM), were observed, indicating that the observed differences in larynx size vs. body mass between primates and carnivorans are robust to differences in the age (RA: *t*_43,39_ =0.959, *P* =0.3435; RA_x_BM: *t*_43,39_ =-0.287, *P* =0.776), sex (SS: *t*_41,37_ =1.429, *P* =0.161; SS_x_BM: *t*_41,37_ =-0.647, *P* =0.521), and sexual dimorphism (SSD: *t*_43,39_ =1.039, *P* =0.305; SSD_x_BM: *t*_43,39_ =-1.036, *P* =0.307) of specimens across our sample. We also conducted a PGLS regression predicting larynx size as a function of BM with SS_x_SSD as a co-variate, assessing a possible effect of specimen sex dependent on species’ sexual dimorphism. The effect of the SS_x_SSD co-variate was not significant (*t*_41,37_ =1.391, *P* =0.173).

*Multi-regime OU evolutionary modelling.* Two significant grade shifts were found in the larynx size vs. body mass allometry across our sample. The first was towards larger residual larynx size in primates, estimated to have occurred at the root of their divergence from carnivorans. The second was towards even larger larynges in *Alouatta*, estimated at the root of their divergence from *Ateles*. Bootstrap analysis further confirmed support for these estimated grade shifts.

*Evolutionary rate comparison.* There was a significant difference between primates and carnivorans in the amount of trait variance in the larynx size to body mass relationships accumulated per unit time (rate ratio =3.31x, *P* =0.00572), reflecting the fact that residual variation of the primate larynx size to body mass allometry was significantly higher than residual variation of the carnivoran allometry (i.e., carnivorans exhibited stronger allometric integration).
