# Supplementary figures and images for: Rapid evolution of the primate larynx?
Source: PLoS Biol. 2020 Aug 11;18(8):e3000764. doi: 10.1371/journal.pbio.3000764 (PMC7418954; doi:10.1371/journal.pbio.3000764)

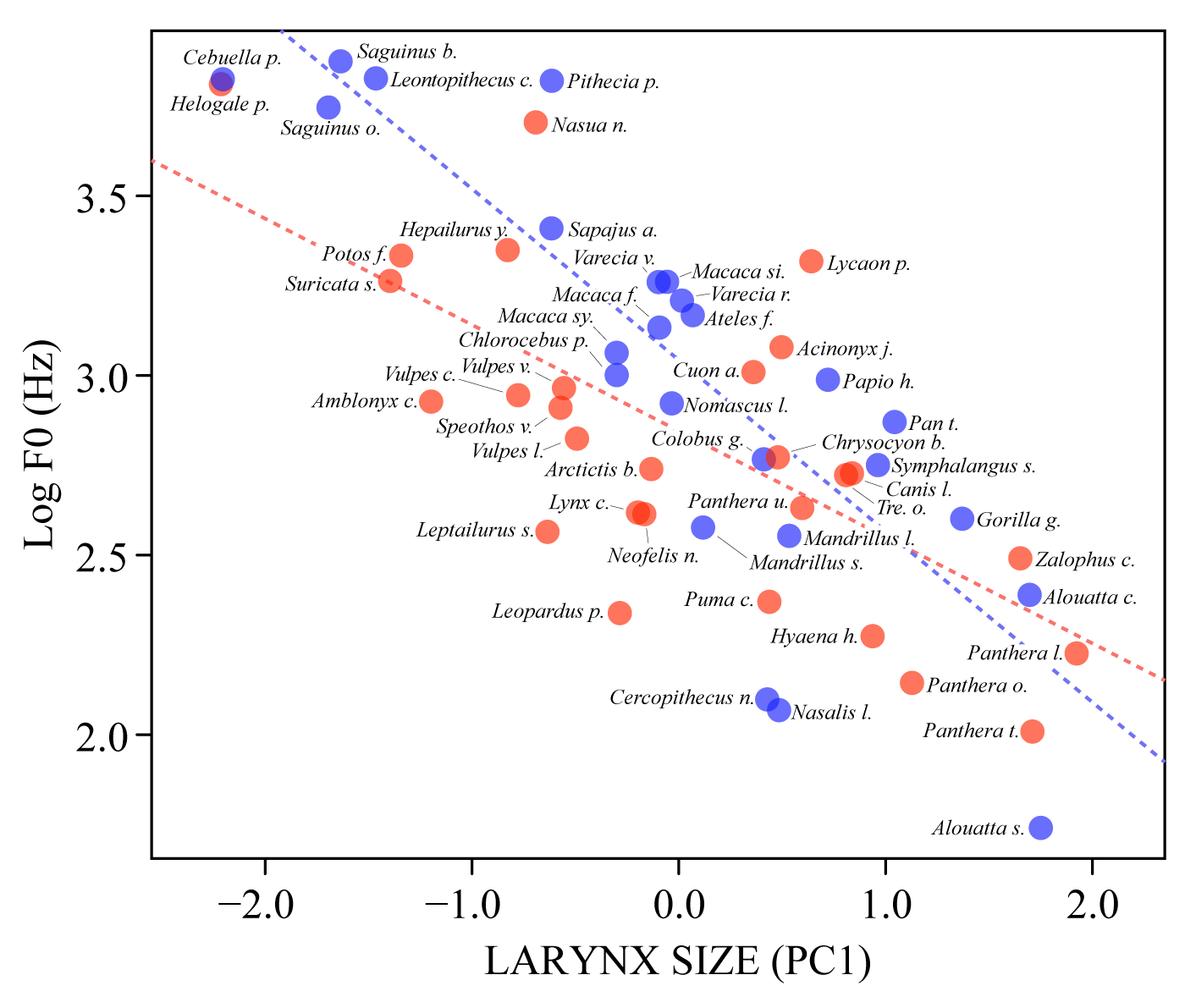

Supplement: S1 Fig — See Fig 3 for full species names. The data used to create this figure are located in S1 Data, sheet B, columns C and K. (DOCX) [file pbio.3000764.s003.docx]

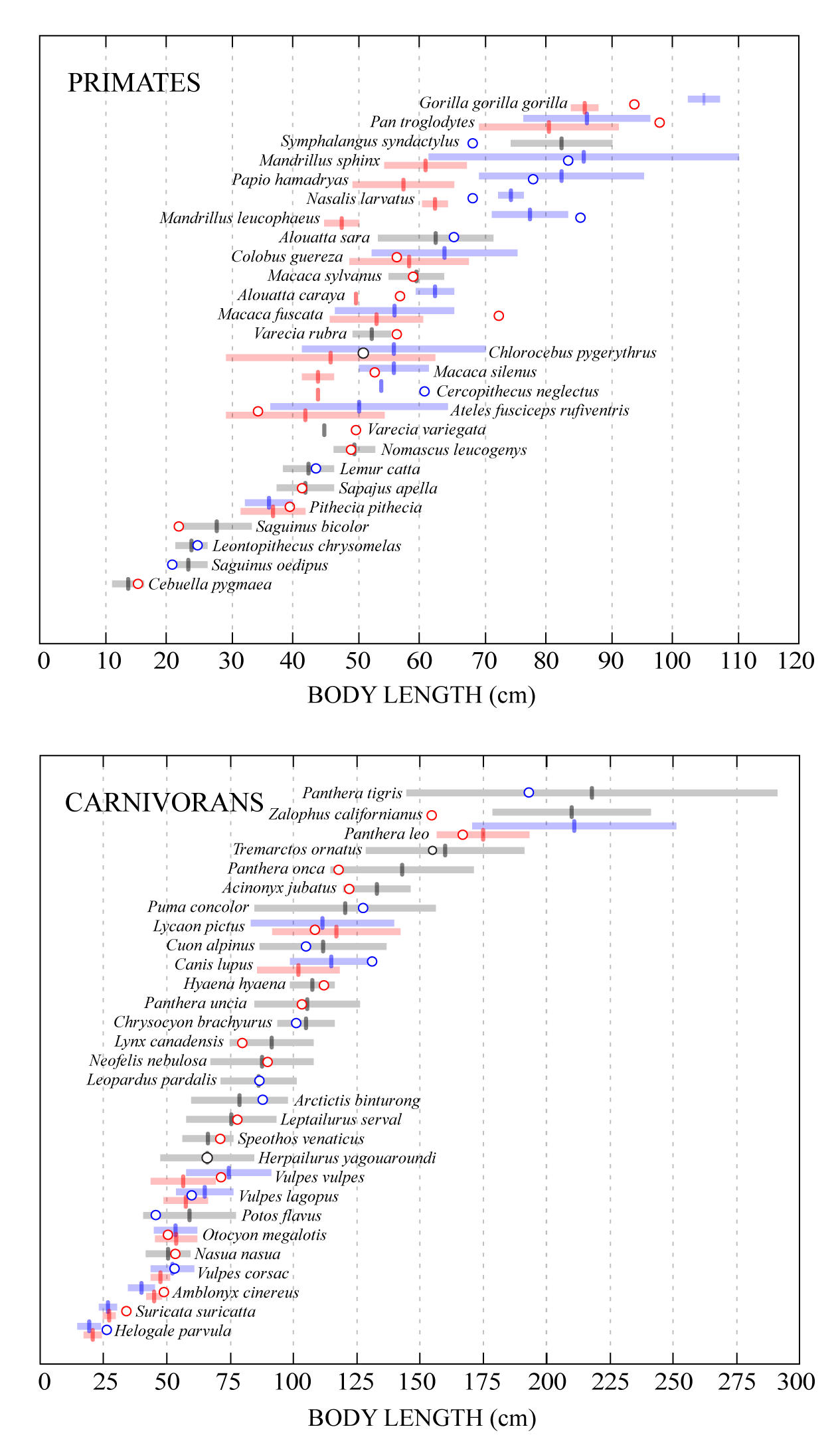

Supplement: S2 Fig — Each circle represents one specimen and is labeled with species name (blue = male, red = female, gray = sex unavailable). Horizontal bars represent body length ranges and vertical bars represent body length means for each species as reported in [46,47]. These ranges and means are given for males (blue) and females (red) separately, when reported separately in [46,47] or in gray when reported together. The four smallest primate specimens and two smallest carnivoran specimens were scanned using micro-CT. The data used to create this figure are located in S1 Data, sheet B, columns B, and G. CT, computed tomography. (DOCX) [file pbio.3000764.s004.docx]
