# Supplementary material for: Rapid evolution of the primate larynx?
Source: PLoS Biol. 2020 Aug 11;18(8):e3000764. doi: 10.1371/journal.pbio.3000764 (PMC7418954; doi:10.1371/journal.pbio.3000764)
Supplement: Attachment — Submitted filename: Bowling et al. PLOS BIOL revision Reviewer Response Letter.docx [file pbio.3000764.s005.docx]

**REVIEWER REBUTTAL LETTER**

Reviewer comments in bold. Responses in plain text.

~~~~~~~~~~~~~~~~~~

**Reviewer #1: In this paper, the authors study the laryngeal morphology of a truly impressive number of carnivorans and primates spanning almost the entire size range of both orders. (I wish they got a least weasel or ermine in there, and the bears too!) Although the findings are rather simplistic - mostly boiling down to 'primates have relatively larger larynges and more size range especially in subclades including howlers with huge ones and papionids with small one - I honestly think that this analysis should be published largely as it. It will be of interest to many of my colleagues.**

**However, there are two minor things that need to be fixed first and one somewhat major thing as well. Namely...**

**1) The title needs to be changed! This paper is about primates AND carnivores. Must say that!**

We are sympathetic to the reviewer’s non-primate-centric concern here. However, we prefer to keep a short title focused on broad implications rather than an explicit description of our results. Thus, we have tempered our original title, turning it into a question rather than a statement. In the discussion, we provide several reasons to believe that primates are indeed the clade with larger, more varied larynges, under faster rates of evolution, and also discuss the need to provide further comparisons in the future to confirm this. We will ensure that both “primate” and “carnivoran” are listed as keywords, and we note that both appear early in the abstract. We hope that this will be agreeable to the reviewer and editor.

**2) Much more care needs to be taken with the scientific names in this paper. There are many capitalization errors (i.e., lowercase genera and uppercase species) and at least one (Cebuella) outdated name. Fix that.**

Thank you for pointing this out. All genera and species errors have been corrected. Biological families and tribes are now capitalized as well, where appropriate (we also use terms such as cebid, papionine, callitrichid, etc., which do not require capitalization).

*Cebuella* has been retained for consistency. The species level information in this paper comes from a single authoritative source (*Handbook of the mammals of the world*). *Cebuella* is also the genus currently used by the International Union for Conservation of Nature.

References:

Mittermeier RA, Rylands A, Wilson DE eds. (2013) *Handbook of the mammals of the world, volume 3: primates* (Lynx Edicions, Conservation International, IUCN, Barcelona).

Wilson DE, Mittermeier RA eds. (2009) *Handbook of the mammals of the world, volume 1: carnivores* (Lynx Edicions, Conservation International, IUCN, Barcelona).

IUCN: <https://www.iucnredlist.org/species/41535/17935567>

**3) The last change is much more substantial, but, in my opinion, essential: the authors MUST rerun their analyses using not only body length (fine to leave that in) but ALSO body mass. That is, the findings would be very different if carnivores are systematically longer than are primates, and, well, carnivores ARE longer-bodied than are primates! I THINK the findings will hold up, but the intercept difference will likely be much less extreme with body mass as a proxy for body size. Both body size proxies should be used and discussed, but without the body mass proxy, people like me might simply say that primates don't have larger larynges, they just have shorter bodies! Prove me wrong.**

**Although this latter request requires rerunning all of the analyses and presenting and discussing a second set of results, it shouldn't be too hard to simply replicate what was done once body masses are gleaned either from the specific data (i.e., vet records) or the literature (often a better place for more homogenous results).**

**Once this is done, I very much look forward to seeing this paper published!**

To address this important point, we have now conducted additional analyses using body mass instead of body length as our proxy of body size. The results strongly support the findings reported in our initial submission. We have briefly described these results in the main text of the paper and included them as supporting information.

The following has been added to the main text (L128-140).

To test whether these results are confounded by using body length as our proxy of body size – e.g., perhaps carnivorans are simply longer-bodied than primates – we repeated the analyses described above using body mass, rather than body length, as our proxy for body size. The results were much the same: primate larynges remained larger and more variable than carnivoran larynges, co-variate effects and their interactions with body mass were not significant, and the average rate of primate laryngeal evolution was significantly greater than the rate of carnivoran laryngeal evolution. The only substantive difference was that in the mass analyses, only one grade shift beyond that between primates and carnivorans was observed: towards larger larynges in *Alouatta*, at the root of their divergence from *Ateles*. Together, the mass analyses indicate that our findings are not an artifact of using length as a proxy for body size. See S1 text for full reporting of the body mass results.

In addition to presenting the mass results with statistical detail, S1 Text includes the following explanation for why we chose length as our primary proxy of body size:

Weights were not available for the majority of our specimens. In most cases “Weight” was given as “PM’d” in the zoo records that accompanied specimens, indicating that a post-mortem autopsy had been performed, presumably without first taking a weight. The analyses using body mass presented below are thus based on species’ averages (from references 44 and 45) rather than the specimen-specific body length measures used in the main text. This, together with the fact that body masses in the zoo often differs substantially from those in the wild, is the reason that we chose length as the main proxy of body size.

~~~~~~~~~~~~~~~~~~

**Reviewer #2: In some ways this is a beautiful study, aiming to understand evolutionary processes on larynx morphology, with a view to gaining insight into selection pressures on vocal communication. The authors compare a number of species from two clades, carnivores and primates, for larynx morphology and body size. They show that larynx size appears bigger in primates relative to body size, with some interesting shifts also within the primate lineage.**

**The problems then come with trying to link larynx size to the fundamental frequency of vocalisations. A major concern here is the quality of the vocalisation data (taken from Bowling et al. 2017), which as far as I can tell does not take into account the sex of the caller, even though many of the species included show substantial sexual dimorphism, nor does it take the huge range of fundamental frequency evident within a number of species into account. Both issues likely produce substantial errors into this regression analysis, as explained in more detail below.**

There are several points to address here.

Perhaps most importantly, please note that the primary aim of our paper is not to produce the best estimate of the regression equation that relates larynx size and F0. Rather, the aim of our paper is to present and analyse a novel and unique database of digitally-modelled scanned larynges from 55 different mammalian species, representing a wide range of body sizes in the primate and carnivoran orders. We use phylogenetic comparative methods to analyse the evolution of this functionally important anatomical trait, highlighting some surprising and, we think, interesting differences between the two clades. At the end of our results section we go on to relate our morphological data with acoustic data as a “sanity check”, to confirm whether our anatomical findings align with the acoustics of vocal behavior, as predicted by current bioacoustic theory. In other words, the point of the acoustic analysis in question is to show that our “larynx size” measure is related to the acoustics of vocal communication, which it clearly is. Acoustics is not otherwise a focus of this paper, e.g., we do not mention it in the abstract.

With respect to data quality, please note that the F0 acoustic allometries reported in Bowling et al. (2017) are the strongest currently available for the orders considered (in the sense of greatest r^2^). The presumptive reason for this is the consistent and systematic approach taken to deriving mean F0 across species in that paper (and by extension this one). Accordingly, mean F0 was determined in precisely the same manner for each of the 53 species included in our acoustic analysis. Original audio was sampled in a way designed to maintain spectral diversity, F0s were calculated on the subset of samples in which it was present, and mean F0 was calculated across this subset. This approach is not without its limitations, but given the number and diversity of species we studied and the lack of consistency in the availability/quality of vocal analyses across them, we consider it to be reasonable to use here.

Criticisms about caller sex and F0 range are addressed below.

**Another major issue is that the authors wish to use their results to derive hypotheses about vocal evolution. However, the hypotheses presented in the discussion are missing a number of references, with the result that some of the come across as somewhat far-fetched. See below for further** **details**.

A total of eight references have been added to the manuscript (see below).

**1. Relating to the regression analysis:**

**P.8 For the regression analysis comparing vocalisations and larynx size across clades, there is no mention of how sex of the signaller was controlled for. As mentioned in the manuscript many species in these clades demonstrate considerable sexual dimorphism. The *Puts et al. 2016* paper cited here also demonstrates substantial sexual dimorphism vocally with males of some species having half the fundamental frequency of females when comparing exactly the same type of vocalisation eg a grunt or scream (all the data for this analysis can be found open access on a dryad and is well worth examining). This manuscript draws its vocal data from the Bowling et al. 2017 paper, which in contrast, as far as I can tell, does not identify the sex of the caller. If this is really the case, then substantial error could be introduced into this regression.**

With respect to the sex of the caller, the reviewer is correct that Bowling et al. (2017) did not include this information – as it was not specified in the audio analyzed (unfortunately it rarely is) – and discuss the fact that this represents a potential source of error in the regression. Including this sex information while maintaining our phylogenetic breadth would be ideal, but it would also require a massive audio recording effort that is beyond the scope of the original paper, and even more so this paper (which is not directly focused on acoustics). Sex labeled vocalization data/audio simply does not exist for most of the species we examine here (especially the carnivorans). Taking *Puts et al. (2016)* as an example, data are only provided for 5 of the 53 species studied here, and they are all primates. Importantly, inclusion of a species here was primarily determined by whether or not we could obtain a viable laryngeal specimen, reflecting the anatomical rather than acoustic focus of our paper.

With regard to the magnitude of the error introduced by not being able to label caller sex, please note that our inclusion of species covering 3 orders of magnitude in body length means that variation across species is considerably greater than variation within any single species (even accepting the fact that some species show sexual dimorphism). This was done intentionally to mitigate the effects of inevitable error arising from only having a few measurements per species.

**Another source of error for this regression is that the vocal repertoire range per species does not seem to be sufficiently accounted for. The authors argue they have used the 'mean F0' per species, a modified version of that used in the Bowling et al. 2017 vocal analysis, using just three calls per species. As far as I can tell, it is not stated whether or not this includes the full range of F0 per species, and when using just 3 calls per species, seems unlikely to be the case. Primate and carnivore vocal repertoires can have a large range of F0, covering > 1000Hz ie covering most of y axis of Fig. 4c. Think about a wolf growl versus a wolf howl.**

Here again, the reason we did not use a “vocal repertoire range” variable is that, for most of our species, acoustically detailed vocal repertoire data do not exist (with carnivorans again being less well represented).

With respect to the variable we did use – mean F0 – please consider the following. Our values were modified from Bowling et al. (2017) by decreasing the number of vocalizations used to calculate species’ means from 6 to 3. We did this because it increased coverage for our sample from 44 species to 53 species (out of 55). Critically, vocalizations were dropped systematically, maintaining in each case the range of F0s present in the original set of 6 vocalizations. Accordingly, the mean F0 values included here are correlated with those in Bowling et al. (2017) at r = 0.975, which suggests that our relatively restricted selections of 3 vocalizations used here are representative of the larger sets of 6 used in Bowling et al. (2017). We additionally note that the paper cited by the reviewer as an example of a superior acoustic analysis – *Puts et al. (2016)* – based their findings on as few as 2 vocalizations per sex per species, highlighting the fact that working with intraspecifically limited data when conducting large-scale interspecific bioacoustic analyses is often a practical necessity in current work in this field, rather than a specific limitation of our work. Finally, with respect to the question of F0 range specifically, we have checked whether this variable explains more variance than mean F0 in our comparisons with larynx size. The results suggest that it does not: the *r^2^* for the LarynxSize vs. log10 meanF0 relationship is 0.583, whereas it is 0.513 for the LarynxSize vs. log10 F0range relationship.

**The extent of the potential error introduced by both of these issues can be illustrated using the recent study of Hammerschmidt and Fischer 2019 J Human Evol, examining the vocal repertoire across 3 baboon species, showing that the baboon vocal repertoire is highly conserved across species. Fig. 1 exemplifies the problem: the spectrograms show that the fundamental frequency in baboon repertoire covers >1500 Hz from grunt to scream. In this manuscript, the authors indicate the hamadryas baboon as having a mean F0 of about 1000 Hz (high above the regression line), whereas the mandrills are shown to have a mean F0 of about 350 Hz (well below the regression line). The Hammerschimdt and Fischer paper show that these two data points are highly unlikely to actually represent the mean F0 for each species, but rather reflect that the three vocalisations used to derive the mean F0 were representative of only part of the total range of F0 for which the larynx was designed.**

Regarding the extent of potential error, please see our replies above concerning the magnitude of variation across vs. within species.

Regarding Hammerschmidt and Fischer (2019), it is not clear to us why this is relevant here. Hammerschmidt and Fischer (2019) examine chacma, olive, and guinea baboons, which, like the hamadryas baboon in our study, are all members of the genus *Papio*. Mandrills belong to the genus *Mandrillus*, which is more closely related to cercocebids than papids. Mandrillids also differ from papids in their ecology, living in dense forests rather than open savanah/woodland/hills, which, according to the acoustic adaptation hypothesis (Ey & Fischer, 2009), creates pressure towards lower F0s in mandrills (as observed here). Further, the mean F0 value we give for the hamadryas baboon (973Hz) is on par with the average that would be expected from the spectrograms in Figure 1 of Hammerschmidt and Fischer (2019) (they do not calculate mean F0). The most extensive paper on the vocal behavior of mandrills provides a thorough list of 10 call types but does not provide quantitative descriptions of F0 (Kudo, 1987), underlining the variation that exists in the quality of quantitative data on vocal acoustics in the literature.

References:

Ey, E. & Fischer, J. The ‘acoustic adaptation hypothesis’ - a review of the evidence from birds, anurans and mammals. *Bioacoustics* **19**, 21–48 (2009).

Kudo, H. The study of vocal communication of wild mandrills in Cameroon in relation to their social structure. *Primates* **28**:289-308 (1987)

**It seems that whether it is the smaller carnivore or larger primate larynxes, the larynx is not designed to produce a limited range in Hz but to produce a diverse range in Hz. Going back to the wolves, whilst short-distance transmission calls, like low Hz growls function to intimidate conspecifics in close proximity, long-distance transmission, but high Hz, howls also likely function to intimidate conspecifics: the selection pressures shaping Hz of vocalisations are likely to be multiplex.**

Agreed. We do not claim otherwise. As explained in the paper, the analysis in question is included as a demonstration that our anatomical findings are related to the acoustics of vocal behavior. That they are, seems to be a reasonable conclusion.

**Thus, until these errors in the vocal data are addressed, it is to me unclear where the regression lines for either clade actually lie.**

Respectfully, providing the best estimate of the location of the regression line is not a goal of this paper. We did not collect/collate any novel acoustic data, and we do not perform any actual acoustic analyses. Instead, we use data from previous work to make a relatively minor point.

**2. Relating to the hypotheses:**

**P. 11. The hypothesis relating to diet differences and choking risk is interesting, however, isn't choking only possible with a descended larynx? Otherwise the epiglottis completely covers the larynx during swallowing (the reason why human babies cannot choke) eg Nishimura, Takeshi. "Comparative morphology of the hyo-laryngeal complex in anthropoids: two steps in the evolution of the descent of the larynx." Primates 44.1 (2003): 41-49.**

It is true that the epiglottis provides critically important protection, but it is false that human babies cannot choke – they can and do (e.g., see Fangupo et al. 2016). But, we take the reviewer’s point and have adjusted the text accordingly (L205-209):

A first possibility is that differences in relative larynx size between primates and carnivorans reflects its role in protecting the respiratory system during feeding (28). Although the trachea is mainly occluded by the epiglottis (not examined here) during swallowing, the larynx protects against aspiration of food or liquid via reflex closure and coughing if the epiglottis is bypassed.

References:

Fangupo, LJ et al. A Baby-led approach to eating solids and risk of choking*. Pediatrics* **138**: e20160772 (2016).

References added to the main text:

Harrison DFN (1995) *The anatomy and physiology of the mammalian larynx* (Cambridge University Press, New York, USA).

**P. 12. Is there any evidence to show that differing rates of oxytocin metabolism would select for variation in larynx size (no references are presented)? Surely selection is much more likely to affect structures where respiration occurs eg at the lung tissue level. The authors themselves argue that it is a weak argument. Why present it then if it has not been substantiated elsewhere?**

Assuming “oxytocin” = oxygen, we are not aware of any evidence linking oxygen metabolism to selection for variation in larynx size.

We do not see a clear logic by which it can be assumed that changes in laryngeal morphology are necessarily less probable than changes in lung tissue given differences in selective pressures related to respiration.

Either way, a respiratory hypothesis has been included here in response to reviews of earlier versions of this manuscript which argued against our greater focus on vocal functions. In response we thought it reasonable to consider all major functions of the larynx in our discussion as potential domains of selection. Although some readers will find some of these hypotheses more/less plausible than others (likely as a function of their backgrounds), our approach serves to map out the space of possibilities, as far as is presently possible and to the best of our ability.

References added to the main text:

Kirchner AJ (1993) The vertebrate larynx: adaptations and aberrations. *Laryngoscope* 103:1197–1201.

Bartlett D Jr. (1989) Respiratory functions of the larynx. *Physiol Rev* 69(1):33–56.

**P. 14. Group sizes have long been noted to be a poor proxy for social complexity, which should be acknowledged. It seems the rationale for including the group size variable is to determine if there are links between social complexity, here defined as primates living in 'larger, more stable groups characterised by strong [social] bonds'. Here, error in this proxy could be reduced by using group sizes for which individual recognition (as an indicator of group stability) between all group members is well established. This would at least require changing the extremely large group sizes noted for sea lions, mandrills and Papio hamadryus. Whilst this may not change the overall result, it would make the analysis more accurate - and hence more compelling - with respect to addressing this particular hypothesis.**

An excellent point. We would love to have data on group sizes for which individual recognition is maintained across our data set. Unfortunately, these data do not exist. We now acknowledge the challenges in measuring social complexity, and the inadequacy of “group size”, with the following text and added reference (L302-305)

These data suggest that social complexity, at least as far as it can be approximated by social group size (see (46) for discussion), is unlikely to explain the clade-wise differences in larynx size that we observe.

References added to the main text:

Bergman TJ, Beehner JC (2015) Measuring social complexity. *Anim Behav* 103:203–209.

**Reviewer #2 (Minor Comments):**

**3. Other points:**

**- The large variation reported in some body length measures should be re-checked such as of Mandrillus sphynx - reaching longer body length of a gorilla, and Pantera tigris varying from 125-300 cm (SI Fig.2)**

These data are from reference 44, and have not been copied in error. Head-body length for the mandrill is given as 62-110 cm in males, whereas the same measurement is given as 103-107 for male Gorillas. Note that the this is not as large as the Tiger (the x-axis of the carnivoran panel is on a different scale). Further, please note that these are only guidelines, and that in many cases, there have not been enough measurements to form very accurate estimates. These estimates are only important here in contextualizing the size of our specimens. Accordingly, we chose to use a single, authoritative text to obtain measurements for all species (*The handbook of mammals of the world*).

References:

Mittermeier RA, Rylands A, Wilson DE eds. (2013) *Handbook of the mammals of the world, volume 3: primates* (Lynx Edicions, Conservation International, IUCN, Barcelona).

Wilson DE, Mittermeier RA eds. (2009) *Handbook of the mammals of the world, volume 1: carnivores* (Lynx Edicions, Conservation International, IUCN, Barcelona).

**- P.14 Last paragraph: large larynxes may be more robust and suited for consistent use. This is a rather vague statement - please make it more specific - and referenced.**

Edited (L292-296):

Our results may be consistent with this hypothesis insofar as the more “complex” social lives of primates can be expected to result in pressure for larger larynges, which could potentially provide a more robust framework matched to the increased mechanical forces expected from more frequent use (41)

Reference added to the main text:

Grodzinsky AJ, Levenston ME, Jin M, Frank EH (2000) Cartilage tissue remodeling in response to mechanical forces. *nnu Rev Biomed Eng* 2:691–713.

**- P.15, Some primates have home ranges that are close to the auditory range of their loudest calls - please provide a reference.**

This point has been dropped from the manuscript for clarity.

**- P.15. More territorial (less social) species - ref missing.**

This point has been dropped from the manuscript for clarity.

**- P.13 'balanced for specimen sex'. Having more species represented by a female than by a male, with no account made of level of sexual dimorphism, and usually only one specimen per species is not the same as 'balanced'. Please modify this accordingly.**

This has been dropped from the manuscript for clarity. Our intention was to reference the fact that our primate and carnivoran samples included very similar numbers of males and females, as specified in the methods. Please also note that specimen sex and level of sexual dimorphism are explicitly modeled in our co-variate analysis.

**- Table 1 shows vocal fold length as measured from 9-12. Please clarify in the table legend that the reference for these landmarks are in Fig. 1. Please also note in the table legend - as well as the figure legend - that 12 is the same as 13 but on the right side (otherwise it is somewhat confusing).**

Done.

~~~~~~~~~~~~~~~~~~

**Reviewer #3:**

**This is an interesting paper on the evolution of the primate larynx. The question is important, and the general approach sound. I commend the authors on putting together an impressive dataset, and for writing such a clear paper.**

**The results show that there is a grade shift between primates and carnivorans in relative larynx size. This isn't especially interesting in itself, but what is much more interesting is the finding that across primates, relative larynx size is more variable than it is in carnivores, and has undergone faster rates of evolution.**

**As far as I can see, the authors have not actually tested whether it is true that primates are more variable? The result is described in the last sentence of the second paragraph of the Results, but there is no analysis presented there to support it.**

This result is supported by a permutation analysis conducted on the primate-to-carnivoran Brownian motion rate ratios. It consists of comparing the Brownian motion rate of evolution of the residual allometric deviation between primates and carnivorans. The Brownian motion rate parameter quantifies accumulation of variation over time, and is thus appropriate for testing the significance of differences in evolutionary rate between primates and carnivorans. In addition, because residual allometric deviation is a direct measure of residual variation, this comparison also addresses the significance of differences in residual variation between primates and carnivorans. The rate ratio and p-value associated with this test have been added to the line in question, along with a brief explanation and a pointer to the methods for more information (L67-69).

**Assuming that it is true that primates are more variable than carnivores, my main comment is that I am unconvinced that it is possible to conclude from this analysis that primate relative larynx size is particularly variable or has undergone particularly fast rates of evolution. It seems to me that it would be equally justified from the presented data to conclude that carnivoran relative larynx size is an especially conserved trait (as the authors note on Pg 15). If the authors were to add say 3-5 more orders, and find that primates have the least variable relative larynx sizes of all tested mammalian orders and with the slowest rate of evolution, bar carnivores, how would that affect their interpretations? It seems to me that the paper would be completely different - concluding that primate relative larynx size was a relatively conserved trait compared to other orders (though not as conserved as what would then appear to be a very conserved carnivoran relative larynx size).**

**Four scenarios seems possible from the present data: 1) primates are more variable and have undergone fast rate changes compared to other mammalian orders (the authors' interpretation); 2) primates are similarly variable and have undergone similar rate changes to other mammalian orders, but carnivore relative larynx size seems constrained (more data needed on other orders); 3) both primates and carnivores are relatively variable and have undergone relatively fast trait evolution compared to other orders, and primates even more so than carnivores (more data needed on other orders); 4) both primates and carnivores are relatively invariant and have undergone relatively slow trait evolution compared to other orders, and carnviores even more so than primates (more data needed on other orders).**

**I realize that collecting data on other orders is a huge amount of work, but it's hard for me to see how it's possible to distinguish between these possibilities with the present dataset. I think that the interpretation of the significance of the difference between primates and carnivores would be completely different depending on which of the above is supported.**

This is the critical point. We have addressed it by removing language from the paper that concludes that primate larynges are definitely exceptional within a broader phylogenetic context (e.g., mammals), AND we have added a “?” to the title to acknowledge this up front.

Unfortunately, our current resources demand that extending this work to include an even more diverse sample remains a goal of our future work. Just assembling the collection of animal remains examined in this paper required nearly 10 years of international collaboration between two institutions and a dozen zoos throughout Europe. Not to mention the time and resources required to excise, transport, scan, model, measure, and analyze the laryngeal specimens. Extending our analysis of primates (N=26) and carnivorans (N=29) to include a comparable number of specimens from addition clades is therefore beyond the scope of this work. Although a broader sample would allow us to draw broader conclusions, we emphasize that our results nonetheless comprise an important advance in understanding the phylogenetic context for laryngeal evolution, and one that is necessary to justify further investment in this research program.

Finally, in many ways carnivorans are a logical starting point to begin large-scale bioacoustic comparisons with primates. They live in a similar range of habitats, body sizes, and social systems. Additional clades that will certainly be interesting to examine using a similar approach to that taken here (e.g., Chiroptera, Rodentia, Artiodactyla), but differences in these life history variables are likely to make bioacoustic comparisons more difficult and less direct.

**In addition, as the authors show, there have been some additional grade shifts among primates, and Alouatta in particular seem very outlying. Does the primate pattern of greater variability hold without Alouatta?**

The statistical analysis presented in Figure 3 indicate that the best phylogenetic model for our primates and carnivorans involves four significant grade shifts, of which atelids are only one (and papionines another). Removing the atelids and papionines from the analysis would constitute artificially homogenizing the data. The fact that primates comprise clades that are significantly different from other primates and carnivorans, whereas carnivorans do not comprise such clades, is informative and biologically relevant. Thus, there does not seem to be any biological or statistical justification for removing these ‘outlier’ groups, which are part of the natural variation.

**I have additional comments on the analysis itself. I didn't find these very clear - the authors should be more explicit exactly how many models were run and how each model was specified.**

We hope to have now made the description of the statistical design straightforward. The methods paragraphs describing it have been thoroughly edited for clarity in accordance with this criticism (L458-526). The essential points are: (1) To estimate if and where grade shifts occur in the bivariate relationship of larynx size and body length we use multi-peak OU modelling. (2) To test whether the grade shifts estimated by the OU modelling result in statistically significant variation among observed tips, we use phylogenetic ANCOVA. (3) To test whether residual variation and rates of evolution are different between primates and carnivorans, we use permutation testing and the Brownian motion rate parameter. These statistical approaches are gold standard in phylogenetic comparative analysis and have been thoroughly validated in previous work.

For example:

Smaers JB, Rohlf FJ (2016) Testing species’ deviation from allometric predictions using the phylogenetic regression. *Evolution (N Y)* 70(5):1145–1149.

Smaers JB, Mongle CS (2018) Evomap: R Package for the evolutionary mapping of continuous traits.

Smaers JB, Mongle CS, Kandler A (2016) A multiple variance Brownian motion framework for estimating variable rates and inferring ancestral states. *Biol J Linn Soc* 118:78–94.

Smaers JB, Mongle CS (2017) On the accuracy and theoretical underpinnings of the multiple variance Brownian motion approach for estimating variable rates and inferring ancestral states. *Biol J Linn Soc* 121(1):229–238.

Revell LJ (2012) phytools: an R package for phylogenetic comparative biology (and other things). *Methods Ecol Evol* 3:217–223.

Adams DC (2014) Quantifying and comparing phylogenetic evolutionary rates for shape and other high-dimensional phenotypic data. *Syst Biol* 63(2):166–177.

Cressler CE, Butler MA, King AA (2015) Detecting adaptive evolution in phylogenetic comparative analysis using the Ornstein-Uhlenbeck model. *Syst Biol* 64(6):953–968.

**In addition: 1) It seems (?) that relative larynx size (residual variance of larynx size against body length) was used as a dependent variable? (Pg 22). If this is the case, analyses should be redone with absolute larynx size as the dependent variable, and body length as a covariate. Using residuals as data is one of Freckelton's (2009) "7 deadly sins of comparative analysis";**

Using covariates in a multiple regression is certainly a valid approach, e.g., we use it here to test for the effects of covariates (sex, sexual dimorphism, and relative age). Critically, however, the use of covariates is not a one-size-fits-all solution to all allometric analyses. Whether or not it is appropriate depends on the research question at hand. Specifically, covariate approaches do not address research questions about evolutionary patterns that underpin bivariate allometric relationships, which are central to comparative biology. In the present manuscript, the questions addressed are fundamentally about the relationship between body size and larynx size. Because bivariate allometric relationships relate to genetic and developmental coordination, shifts in the allometry can be considered as shifts in the underlying genetic and developmental regulation of the bivariate system. A covariate approach is not relevant in this context, because the questions are focused on bivariate relationships, i.e., they pertain to the two variables in question, and how they relate, rather than potential covariates.

In order to capture the precise bivariate relationship between two variables, it is fundamental to check whether the allometry is monotonic (of single intercept and single slope). The standard way in which to do this, is to analyze residual deviations. In fact, *anything* in regression analysis pertains to analyzing residual deviations. If using residual deviations were invalid, regression analysis itself might be considered invalid.

The citation highlighted by reviewer #3 argues that the problem with using residuals as data is that there may be covariates that confound the bivariate relationship. This is particularly relevant in phylogenetic comparative studies of behavior (where confounding effects are very hard to ascertain). However, when dealing with anatomical variables (as here), neither the confounding effects or the research questions are the same. There is no doubt that size is a critically important factor in biology, and it is not clear, even in theory, what kind of covariate could confound it here. Likewise, our research questions are focused on identifying the evolutionary patterns that characterize the bivariate relationship between two size variables. This is central to the study of allometry. The question of what covariates might confound evolutionary studies of behavior is thus very different from the question of what evolutionary pattern underpins the bivariate relationship of between larynx and body size. These differences require very different approaches.

We use covariates as an appropriate way to check whether sex, sexual dimorphism or relative age are confounding variables (we find they are not). However, when we address our main questions pertaining to the evolutionary pattern that underlies the bivariate relationship of two anatomical variables, we do so using multi-peak OU modelling and pANCOVA, methods designed specifically to address questions about bivariate allometries directly.

Recognition of the fact that using residual variation in allometries between anatomical variables is an effective way to quantify the evolutionary patterns that underpin bivariate allometries can be found in the numerous papers by leading researchers in comparative biology (some of whom are colleagues of Rob Freckleton) that use this exact approach. See for example, the work by Liam Revell and others on lizards (cited below). These authors did not simply ignore Rob’s 2009 paper. Rather, they address different types of research questions than Rob’s critique was targeted towards, and this is also true of our study.

References:

DL Mahler, T Ingram, LJ Revell, JB Losos. (2013) Exceptional convergence on the macroevolutionary landscape in island lizard radiations. *Science 341* (6143), 292-295

LJ Revell, LJ Harmon, DC Collar (2008) Phylogenetic signal, evolutionary process, and rate. *Systematic biology 57* (4), 591-601

DL Mahler, LJ Revell, RE Glor, JB Losos (2010) Ecological opportunity and the rate of morphological evolution in the diversification of Greater Antillean anoles. *Evolution 64* (9), 2731-2745

**2) Log-transformation of data to achieve normality of variable distributions. The models used by the authors do not assume that variables (even dependent variables) are normally-distributed, only that the error structure (residual variance of the model) is. The authors should run their models without transforming variables and check the model diagnostics. Only if the models fail these should the authors consider transforming data or specifying other error structures;**

There are many reasons to log-transform data that extend beyond issues of normality. Most critically here, regression analysis quantifies variation on a linear scale. Size, however, changes on a logarithmic (proportional) scale. When logging size, the linear analysis of (now logged) size gives valid results (i.e., the linear increments on a log scale represent proportional changes). By contrast, when variables that change on a logarithmic scale (like size) are treated and analyzed on a linear scale, the results are not valid.

**3) phylogenetic uncertainty - I couldn't find information about which phylogeny was used? I would encourage the analyses to be run across multiple phylogenies to determine how robust the results are to phylogenetic uncertainty;**

Apologies for this oversight – they were unintentionally removed in previous revisions. The primate tree comes from Nyakatura et al (2012), the primate tree comes from Arnold et al (2010). Both refs are now included on lines 464-466 at the beginning of the methods section on phylogenetic comparative methods.

References added to the main text:

Arnold C, Matthews LJ, Nunn CL (2010) The 10kTrees website: a new online resource for primate phylogeny. *Evol Anthropol* 19(3):114–118.

Nyakatura K, Bininda-emonds ORP (2012) Updating the evolutionary history of Carnivora (Mammalia): a new species-level supertree complete with divergence time estimates. *BMC Biol* 10:1–31.

Regarding phylogenetic uncertainty, previous studies quantifying evolutionary patterns (which is done using OU modelling) have demonstrated that the effect of phylogenetic uncertainty is negligible when effect size is high (see Cressler et al., 2015, cited above). Our effect size is very high (signal-to-noise ratio of 7.29; when SNR>>1 effect size is high) for the evolutionary pattern identified as underpinning the larynx size to body length bivariate space. The fact that, effect size, not phylogenetic uncertainty, is by far the most important factor in such modelling analyses has been validated using exactly the same models used in previous studies (e.g., Cressler et al. 2015).

Of course, this does assume that we are certain enough in the phylogeny to consider it sensible, but the phylogenies of these mammalian clades are well resolved and differences among individual trees are minor. Considering that our results reveal differences among taxonomically relevant clades (atelids versus papionines versus other primates versus carnivorans), not within, it would be unreasonable to suggest that phylogenetic uncertainty would change the results at this level. After all, phylogenetic uncertainty is not so great as to mistake a macaque from a spider monkey or a small cat from a big cat.

**4) Did the authors set lambda to 1, or allow it to take its maximum likelihood value? What was the probability distribution of lambda? In my experience in small comparative datasets such as these, the lambda probability distribution functions are sometimes extremely flat and broad, such that low phylogenetic signal, high phylogenetic signal, and everything in between, are almost equally likely, even though the ML lambda is at one particular end of the distribution function. More generally, unless I missed something I didn't find there to be sufficient information in the manuscript to fully evaluate the comparative analyses.**

We allowed the pGLS to take the maximum likelihood value. The likelihood surface of lambda is not flat for our analyses:

Unsurprisingly given that lambda is estimated to be 0.96, fixing lambda to unity did not alter the results.

**I hope that my comments are helpful to the authors and congratulate them on their interesting work - James Higham**

Thank you James. Your constructive criticism is much appreciated and we have done our best to address it within the scope of this paper.

~~~~~~~~~~~~~~~~~~

**Reviewer #4:**

**I have reviewed a different version of this manuscript previously and I was supportive of its publication at that time. I think it is an interesting study that will lead to new lines of research. I do not have any major issues at this point (my concerns with a previous version have been addresses). One suggestion would be to explore the atelids a bit more as they seem largely responsible for the higher variability in primates.**

Indeed, the atelids remain an interesting case. We cite Dunn et al. (2015) to provide more information, but unfortunately for the present analysis, we were only able to include 2 howler monkey larynges.

**Reviewer #4 (Minor Comments):**

**Page 5. It seems that there should be a test associated with this statement: "Importantly, we also found that residual variation of the primate allometry was significantly higher than residual variation of the carnivoran allometry"**

This result is supported by a permutation analysis conducted on the primate-to-carnivoran Brownian motion rate ratios (see above). The rate ratio and p-value associated with this test have been added to the line in question, along with a brief explanation and a pointer to the methods for more information (L67-69).
