# Supplementary material for: Rapid evolution of the primate larynx?
Source: PLoS Biol. 2020 Aug 11;18(8):e3000764. doi: 10.1371/journal.pbio.3000764 (PMC7418954; doi:10.1371/journal.pbio.3000764)
Supplement: Attachment — Submitted filename: PloS Biol revision3 Response Letter.docx [file pbio.3000764.s006.docx]

**RESPONSE LETTER**

Reviewers comments in bold, responses in plain test.

*Rev. 3: James Higham - please note that this reviewer has waived anonymity*

**I have read the revised version of the manuscript, which I originally reviewed for eLife.**

**I think that the manuscript is much improved. In particular, I found the description of the analyses much clearer. The manuscript is now more appropriately caveated as a comparison of two taxa. I think the manuscript and data presented are interesting, and it was clearly a lot of work, for which I congratulate the authors.**

Thank you! We sincerely appreciate your constructive criticism.

**A couple of remaining comments:**

**1) Now that I can more clearly understand the analyses that were undertaken, I agree with the authors that the analyses are appropriate as they are. However, I'm confused by the authors' response to my comments about log-transformation of variables. In their response, the authors write: "There are many reasons to log-transform data that extend beyond issues of normality." I certainly agree with this. However, I was not the one who suggested that the data were log-transformed to achieve normal distributions of the variables - this is what the authors themselves use as their justification in their manuscript. In the present version of the MS, they write: "Log-transformations (base-10) were necessary to achieve normality (as indicated by Shapiro-Wilk tests) for all laryngeal measures and body length." (Lines 444-446). If the authors have other reasons for log-transforming all their data, then they should delete this and give a different justification. Otherwise, I don't see that they have addressed my original comment.**

Thank you for repeating this point. The Shapiro-Wilk tests and log-transformations were conducted at a very early stage of this work. While it is true that the laryngeal measures and body length were not normal by this standard, you are certainly correct that this is not the most relevant point to make regarding our modelling of them. A more appropriate rationale for the log-transforms is now provided in place of the text you quoted (now on L509-512):

“Laryngeal and body length measures were log-transformed (base-10) because these variables change on a proportional scale whereas our linear models quantify variation on a linear scale.”

**2) The caveats to the two-taxon comparison have improved the manuscript substantively. The authors write: "we cannot be certain whether the primate larynx is exceptionally large and variable or the carnivoran larynx exceptionally small and constrained." (Lines 308-309) The authors should change the word "exceptionally" to "relatively". This is what they have shown - that primates are relatively large and more variable compared to carnivores, and vice versa. For all we know both could be relatively invariate compared to many other mammalian orders, or both could be relatively variable compared to many other mammalian orders. In addition to changing 'exceptionally' for 'relatively', I also think that this latter point should be made explicit at this point in the manuscript.**

“exceptionally” has been changed to “relatively” in both instances (now L367-368).

Your point has also been made more explicit with the addition of the italicized text below (L370-374): “…But settling this issue will ultimately require more context from additional comparisons, e.g., with Artiodactyla, Rodentia, and/or Chiroptera, where Hammer-headed bats provide another known example of laryngeal hypertrophy [43]. *Only such comparisons can tell us how the variability observed here ranks among other mammalian orders.*

**I hope that my additional comments are helpful - James Higham**

Very much so. Thank you James.

- - - - - - - - - - - - - - - - - - - - - - - - - - - -

*Rev. 5:*

**In the same way as all four former reviewers from eLife, I believe that this paper is very interesting and would constitute a very valuable addition to the literature. The data combined in these analyses are very impressive (including 26 primates and 29 carnivorans, collected over 10 years) and the results relatively straightforward. There are also some obvious limitations, as for any analysis that relies on data available online or published in books or by other authors (e.g. exact body size and F0 of the various species included here), in addition to the comparison of 'only' two orders (primates and carnivores). However, I believe that the results presented in this paper deserve to be published, since although the best estimates of the location of the regression lines shown might be revised later on when more exact data about each species is gathered, the results are fairly robust and their publication have the potential to stimulate research in this field.**

Thank you for this fair assessment.

**Since the former reviewers already raised many concerns and I think that the authors responded to these comments very well, I only have a few suggestions to improve the analyses and make the claims stronger.**

**L80-86. I know that the effects of these covariates is not significant in terms of p-values. However, they might still explain some variance in the data, especially for those that show lower p values (e.g. SS). Since potential systematic differences between primates and carnivorans in the sex and age of your individual larynx specimens is one of the major limitation of the study (also highlighted by former reviewers), would it not make sense to run your pANCOVA on the residual variance of larynx size against body length and these covariates (at least SS)?**

We are sympathetic to the reviewer’s desire to see systematic differences between primates and carnivorans in the sex and age of our specimens incorporated into our main pANCOVA analyses of body length vs. larynx size. However, we believe that the following reasons provide a strong rationale for not doing so:

(1) The co-variate analysis that includes the implicated factors shows that they do not explain a significant amount of variance in the body length vs. larynx size relationship, making it unclear what the basis for including them in the pANCOVA would be.
(2) There are no systematic differences in specimen sex (SS), relative age (RA), and species sexual dimorphism (SSD) between our primate and carnivoran samples.

-SS: primates [15f, 10m, 1 unknown] vs. carnivorans [16f, 11m, 2 unknown] (see L322)

-RA: *M_primate_* =0.457 *M_carnivoran_* =0.519, *P* =0.2886 (Mann-Whitney U-test).

-SSD: *M_primate_* =1.029 *M_carnivoran_* =1.024, *P* = 0.5328 (Mann-Whitney U-test)

(3) Including SS, RA, and SSD as co-variates in the pANCOVA analysis would reduce our sample size (since we were not able to obtain all co-variates for all specimens), while adding to the complexity of the analysis with no clear benefit

**L210-211. 'relaxation of selective forces related to diet'. Can you give more details here about which kinds of diet can lead to a relaxation of selective forces?**

The following sentence has been edited and now reads (L269-272): “For example, it may be that the threat of choking on large pieces of minimally-chewed meat leads to selection pressure for smaller larynges in carnivorans, and that this pressure is somewhat relaxed in primates, which eat more plant material and spend more time chewing as a group [29]”

Added reference:

29.	Hiiemae KM, Crompton W. Mastication, food transport, and swallowing. Functional vertebrate morphology. Cambridge, MA: Belknap Press; 1985.

**L205-257. Several of the former reviewers highlight the 'simplicity' of the papers and its results, which are also 'only' based on two mammalian orders. I am thus wondering if the paper could not be substantially improved by actually testing these hypotheses mentioned in the discussion (diet, locomotor behaviour (terrestrial versus arboreal), and habitat (in relation to vocalisations))? Are data available on the diet and ecology of all of these species (or a subset at least) not available? If yes, the authors could maybe test which of these hypotheses explain the variation observed in larynx size the best. If this is not feasible, a general conclusion with the opinion of the authors concerning which of these hypotheses is more likely would be appreciated.**

Throughout the review process we have had to balance our own bias towards a vocal account of our findings with the desire of some reviewers’ to see an even-handed description of alternative possibilities. We believe that what we have now – a discussion that considers explanations based on all major functions of the larynx, presented alongside an abstract, introduction, and conclusion that focus on vocal communication – strikes this balance to a reasonable degree. We are sympathetic with the reviewer’s desire for us to take a stance on this issue, but after having been criticized for doing so previously, we feel that we have said what we can on the basis of our data, and that going further would not add any empirical clarity.

**L296-298. Please provide the statistical results related to these claims.**

Added (now line 356-357).

*Data and other policy-related requests.*

**You mention that all mass data are compiled from references 44 and 45, and that is fine, but if you processed the data and used new derivatives for your figures then you will need to supply the numerical data underlying the graphs. Also, I can see that you have provided a data file (S1 data), so if this data has been used to make the figures, please specify which ones.**

Done.

**Please ensure that you provide the individual numerical values that underlie the summary data displayed in the following figure panels as they are essential for readers to assess your analysis and to reproduce it: Fig. 2, Fig. 3A, Fig. 4B, C; Fig. S3 and Fig. S4**

Done.

**Please also ensure that figure legends in your manuscript include information on WHERE THE UNDERLYING DATA CAN BE FOUND,**

Done.

**and ensure your supplemental data file/s has a legend.**

Done.

**Please ensure that your Data Statement in the submission system accurately describes where your data can be found.**

Done.
